# GPS-Based Exposure to Neighborhood Deprivation and Late-Life Cognition

**DOI:** 10.1093/geroni/igaf122.1888

**Published:** 2025-12-31

**Authors:** Jinshil Hyun, Nelson Roque, Mindy Katz, Richard Lipton, Carol Derby, Charles Hall

**Affiliations:** Albert Einstein College of Medicine, Bronx, New York, United States; The Pennsylvania State University, University Park, Pennsylvania, United States; Albert Einstein College of Medicine, Bronx, New York, United States; Albert Einstein College of Medicine, Bronx, New York, United States; Albert Einstein College of Medicine, Bronx, New York, United States; Albert Einstein College of Medicine, Bronx, New York, United States

## Abstract

Prior literature shows deleterious effects of neighborhood deprivation on cognitive and brain health. Most studies focus on neighborhood deprivation within individuals’ residential areas, but people often travel across multiple neighborhoods. The use of GPS technology allows us to track person-specific exposure to deprived areas as individuals navigate their everyday environments. The aim of this study was to examine associations between GPS-derived exposure to deprived areas and different domains of cognition in late life. Participants include 126 older adults from Bronx, NY enrolled in the Einstein Aging Study (mean age=76; 63% females; 37% non-Hispanic Blacks). GPS signal was measured every minute for up to two weeks. GPS coordinates were coded at the census block group level and linked to publicly available Area Deprivation Index (ADI) scores. The final variable of interest was time-weighted exposure to deprived areas across entire study days for each participant (range = 0 to 9). Five cognitive domains (episodic memory, language, executive function, processing speed, visuospatial) were derived by averaging a subset of scores from 13 standard neurocognitive tests, with final scores in T-score units. Results from linear regression indicated that greater exposure to deprived areas in everyday life was associated with lower levels of executive function (estimate= -1.52, p=.007) and language ability (estimate= -1.41, p=.012) after controlling for age, sex, race/ethnicity, education, and total number of GPS collection days. We did not find significant associations between residential-level ADI and cognition. Everyday exposure to neighborhood deprivation, beyond residential deprivation, may influence cognitive health in older adults.

